# Associations of self-reported height loss and kyphosis with vertebral fractures in Japanese women 60 years and older: a cross-sectional survey

**DOI:** 10.1038/srep29199

**Published:** 2016-07-06

**Authors:** Mikio Kamimura, Yukio Nakamura, Noriyuki Sugino, Shigeharu Uchiyama, Masatoshi Komatsu, Shota Ikegami, Hiroyuki Kato, Akira Taguchi

**Affiliations:** 1 Center for Osteoporosis and Spinal Disorders, Kamimura Orthopaedic Clinic, 595-17 Kotobuki, Matsumoto 399-0021, Japan; 2 Department of Orthopedic Surgery, Shinshu University School of Medicine, 3-1-1 Asahi, Matsumoto 390-8621, Japan; 3 Department of Oral and Maxillofacial Radiology, School of Dentistry, Matsumoto Dental University, 1170 Gobara Hirooka, Shiojiri 399-0781, Japan

## Abstract

Some vertebral fractures come to clinical attention but most do not. This cross-sectional survey aimed to clarify the associations of self-reported height loss and kyphosis with vertebral fractures. We enrolled 407 women aged 60–92 years who visited our orthopaedic clinic between June and August 2014 in our study. Inclusion criteria were lateral radiography followed by completion of a structured questionnaire in this study. The primary outcome was vertebral fracture diagnosed on lateral radiography and graded using a semiquantitative grading method, from SQ0 (normal) to SQ3 (severe). Self-reported kyphosis was defined as none, mild to moderate, severe. Self-reported height loss was defined as <4 cm or ≥4 cm. Number of SQ1 fracture was associated only with kyphosis. Self-reported severe kyphosis was significantly associated with increased numbers of ≥SQ2 vertebral fractures (p = 0.007). Height loss ≥4 cm was significantly associated with increased ≥SQ2 grade fractures (p < 0.001). Odds ratios (ORs) for fractures associated with mild-to-moderate and severe kyphosis were 2.1 [95% confidence interval 1.4 to 3.3) and 4.2 (1.8 to 9.5), respectively. OR for fractures associated with height loss ≥4 cm was 2.3 (1.4 to 3.7). Self-reported kyphosis may be useful for identifying Japanese women aged ≥60 years who have undetected vertebral fractures.

Osteoporosis (OP) is one of the most common metabolic bone disorders worldwide. It has been defined as a skeletal disorder characterized by compromised bone strength, predisposing a person to increased risk of fracture. Bone strength primarily reflects the integration of bone density and bone quality^1^. Bone fragility fracture is considered a complication of OP^2^. Prevention of bone fragility fractures is the primary therapeutic goal when individuals have OP. Although bone mineral density (BMD) measured by dual-energy X-ray absorptiometry (DXA) is usually used for diagnosing OP, the Japanese 2015 OP guideline states that when a fragility fracture (e.g., a proximal femoral fracture or vertebral fracture) is present, the patient is diagnosed as having OP regardless of their BMD values^3^.

Vertebral fracture is the most frequent of these fragility fractures in Japan. The vertebral fracture rate is higher in Asians than in Caucasians^4^. In addition, among Asians, the prevalence of vertebral fracture was highest in the Japanese^5^. Because the presence and number of vertebral fractures are associated with mortality^6^, Japanese elderly populations who have vertebral fractures should be referred to medical professionals and diagnosed as quickly as possible.

Ross reviews that some patients with vertebral fractures experience intolerable pain, whereas about half of all patients with radiographically identified vertebral fractures had no symptoms^7^. Additionally, Vogt *et al*. describe that approximately two thirds of women with radiographic evidence of vertebral fracture are unaware of the fracture^8^. These suggest that despite the high prevalence of vertebral fracture, many vertebral fractures remain undiagnosed. Vogt *et al*. finally developed 5 self-reported simple items to identify women 55 years and older who were likely to have undiagnosed vertebral fractures. Kendler *et al*. recently reviewed that the recognition of vertebral fractures in imaging reports obtained for purposes other than the investigation for vertebral fracture in a hospital setting is generally poor^9^. They also summarize that risk of future vertebral fracture increases with increasing number and severity of prevalent symptomatic and asymptomatic (morphometric) vertebral fractures. Although both asymptomatic (morphometric) and symptomatic vertebral fracture can be diagnosed using the Genant semiquantitative method^10^, we should carefully assess vertebral fractures on lateral radiographs in patients who have symptoms like back pain if simple items imply the possibility of having a fracture. Further, if simple items imply the possibility of having a vertebral fracture, we should take lateral radiographs even in asymptomatic patients, especially when considering a large number of patients.

Kyphosis and height loss, which is determined by comparing height at the time of measurement with that at a younger age, are considered useful surrogate markers or screening tools for vertebral fractures in older people^11^,^12^. Several thresholds for height loss have been suggested to identify individuals who have asymptomatic vertebral fractures^13^,^14^,^15^,^16^. Because the spine is frequently involved, elderly women with kyphosis due to multiple compression fractures are common^17^. After adjustment for age, a 15° increase in kyphosis was associated with the presence of a vertebral fracture [odds ratio (OR) 1.57]^18^. However, there is no available methodology to diagnose kyphosis simply. Since it is likely that patients with undiagnosed vertebral fractures may feel kyphosis themselves, it is very important for us to demonstrate whether simple self-reported kyphosis is associated with the presence of vertebral fractures determined by lateral radiographs.

We hypothesized that simple self-reported kyphosis may be associated with the presence of vertebral fractures in Japanese women aged ≥60 years. The aim of this study, therefore, was to clarify the associations among self-reported kyphosis, self-reported height loss calculated according to the difference in current height and self-reported height at a younger age, and the presence and number of vertebral fractures that were detected on lateral radiographs in older Japanese women.

## Materials and Methods

### Subjects and determination of vertebral fractures

Among patients aged ≥60 years who visited our orthopaedic clinic during June through August 2014, the women were invited to complete a structured questionnaire. Women who refused to provide written informed consent or who did not undergo lateral spine radiography ahead of completing a structured questionnaire examination were excluded from the study. Women who had many missing data in their questionnaire were also excluded from the study. Lateral radiographs of the spine were evaluated for the presence of OP and/or spondylosis. Unfortunately, the precise duration between the radiographs and the questionnaire were unknown. However, women underwent lateral radiographs within a few years before the questionnaire. The subjects completed a structured questionnaire aimed at collecting information regarding self-reported kyphosis, self-reported height at a younger age, history of current and previous smoking, and history of diabetes mellitus, steroid use, rheumatoid arthritis, and use of OP medications. Self-reported kyphosis was simply defined using three categories: none, mild to moderate, and severe. Height loss was calculated according to current height and self-reported height at a younger age and was divided into two categories according to a previous study^16^: <4 cm or ≥4 cm.

Two experienced orthopaedic surgeons, blinded to the responses in the questionnaires, independently determined the presence of vertebral fractures on lateral spinal radiographs using a semi-quantitative (SQ) method developed by Genant *et al*.^10^. With this SQ method, thoracic and lumbar vertebrae are graded by visual inspection of lateral spinal images, generally without direct vertebral measurements. The grades were as follows: SQ0, normal; SQ1, mildly deformed (approximately 20–25% reduction in anterior, middle, and/or posterior height and 10–20% reduction of the projected vertebral area); SQ2, moderately deformed (approximately 25–40% reduction in anterior, middle, and/or posterior height and 20–40% reduction of the projected vertebral area); SQ3, severely deformed (approximately ≥40% reduction in anterior, middle, and/or posterior height and in the projected vertebral area). Uemura *et al*. reported that assessment of vertebral fractures using the SQ method tended to be overestimated by inexperienced physicians compared with those done by experts. For example, the assessments evaluated in that study showed poor non-expert interobserver reliability but well-matched expert interobserver reliability^19^. Because experienced orthopaedic surgeons are experts in assessing vertebral fractures, it is likely that the surgeons in the present study diagnosed the vertebral fractures accurately.

We then counted the number of SQ1 and ≥SQ2 fractures. If the results were different between two observers, consensus was reached by discussion. The Ethics Committee of Matsumoto Dental University reviewed and approved the study protocol. The methods were carried out in accordance with the relevant guidelines, including any relevant details. Written informed consent was obtained from participants.

### Data analysis

The data for continuous variables were expressed as means ± standard deviation (SD). Multiple regression analysis in a stepwise manner—adjusted for age, height, weight, height loss category, and history of smoking (yes or no), diabetes mellitus (yes or no), steroid use (yes or no), rheumatoid arthritis (yes or no), and use of OP medications (yes or no)—was used to clarify the association between self-reported kyphosis and the number of SQ1 and ≥SQ2 vertebral fractures. Dummy variables were used for categorical data in this multiple regression analysis.

Logistic regression analysis with the stepwise forward selection method—adjusted for age, height, weight, height loss, history of smoking (binary), diabetes mellitus (binary), steroid use (binary), rheumatoid arthritis (binary), and use of OP medications (binary)—was used to calculate the odds ratios (ORs) and 95% confidence intervals (CIs) of the presence of fractures (SQ1, ≥SQ2, all fractures) according to the self-reported kyphosis category. Stepwise forward selection used in this study is the method with entry testing based on the significance of the score statistic (p ≤ 0.05), and removal testing based on the probability of the Wald statistic (p ≥ 0.10). The data were analysed using the Statistical Package for the Social Sciences (SPSS, version 19.0; IBM Inc., Armonk, NY, USA). Values of p < 0.05 were considered to indicate statistical significance.

## Results

**Figure 1:**
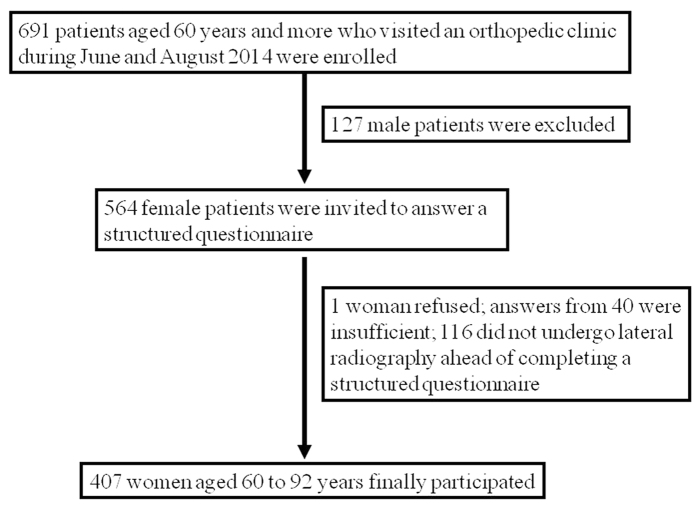
Flow diagram of the subjects included in this study.

Among the 691 patients aged ≥60 years who visited our orthopaedic clinic during June through August 2014, 127 men were excluded from this study. A total of 564 female patients were invited to complete a structured questionnaire examination. Among them, only one woman refused to answer questions on the questionnaire. Although 563 women answered a structured questionnaire examination and provided written informed consent, the answers from 40 of the women were insufficient for the analysis. Many missing or unclear data of the questionnaire were observed in these 40 women. Among the remaining 523 women, 407 aged 60–92 years who underwent lateral spine radiography before completing the structured questionnaire comprised the final participants in this study (Fig. 1). The time interval between the most recent lateral radiograph and the structured questionnaire examination varied among the subjects.

The characteristics of the study subjects are shown in Table 1. In all, 151 subjects reported no kyphosis, 207 had mild to moderate kyphosis, and 49 had severe kyphosis. One-hundred and forty-four (35.4%) subjects had SQ1 fractures. Of these, 78 subjects had 1 fracture, 48 had 2, 12 had 3, 5 had 4, and one had 5 fractures. One-hundred and twenty-seven (31.2%) subjects had ≥SQ2 fractures. Of these, 73 had 1 fracture, 27 had 2, 13 had 3, 8 had 4, 4 had 5, one had 6, and one had 7 fractures. Overall, 217 subjects (53.3%) had fractures ≥SQ1 grade. Almost all of the subjects (89.9%) were taking some OP medication(s) at the time of answering the questionnaire. The mean self-reported height loss was 3.4 cm for the study subjects, and 139 (34.2%) subjects had height loss of ≥4 cm. Table 2 shows the association between self-reported kyphosis, self-reported height loss, and fracture status.

Multiple regression analysis revealed that the self-reported no kyphosis was significantly associated with a decreased number of both SQ1 (p < 0.001) and ≥SQ2 (p = 0.007) fractures (Table 3). Self-reported severe kyphosis was significantly associated with an increased number of ≥SQ2 fractures (p = 0.025). Aging was significantly associated with an increased number of SQ1 fractures (p = 0.010). Increase of self-reported height loss was significantly associated with an increased number of ≥SQ2 fractures (p < 0.001). Use of OP medication (p = 0.001), greater height (p = 0.009), and steroid use (p = 0.025) were significantly associated with fewer ≥SQ2 fractures. *R*^2^ was much smaller for SQ1 fractures than for ≥SQ2 fractures.

Logistic regression analysis with stepwise forward selection after adjusting for the covariates indicated that the ORs for having vertebral SQ1 fractures associated with self-reported mild-to-moderate and severe kyphosis were 2.8 (95% CI, 1.7 to 4.4) and 3.6 (95% CI, 1.8 to 7.1), respectively (Table 4). The ORs for having vertebral ≥SQ2 fractures associated with mild-to-moderate and severe self-reported kyphosis were 1.8 (95% CI, 1.0 to 3.1) and 3.5 (95% CI, 1.6 to 7.7), respectively. The ORs for having any vertebral fractures associated with mild-to-moderate and severe self-reported kyphosis were 2.1 (95% CI, 1.4 to 3.3) and 4.2 (95% CI, 1.8 to 9.5), respectively. Additionally, the ORs for having vertebral ≥SQ1 fractures or any-grade fractures associated with self-reported height loss (≥4 cm) were 3.2 (95% CI, 1.9 to 5.3) and 2.3 (95% CI, 1.4 to 3.7), respectively. Use of OP medications contributed to a significantly decreased risk of having vertebral ≥SQ2 fractures.

## Discussion

This study showed that simple self-reported kyphosis and height loss were significantly associated with the presence and number of vertebral fractures in elderly Japanese women. This finding suggests that these self-reported indices, especially simple self-reporting of kyphosis, are useful for identifying Japanese women aged ≥60 years who have undetected vertebral fractures.

There have been numerous reports on physical findings and risk factors for OP, including low weight, low height, spinal fracture, and skeletal BMD, especially in postmenopausal women^20^,^21^,^22^. Several thresholds for height loss have been suggested to identify individuals who have asymptomatic (morphometric) vertebral fractures. Some reports indicated that a 2- to 4-cm height loss is a clinical sign of spinal fracture^13^,^15^. We observed a significant association between self-reported height loss ≥4 cm and increased risk of vertebral fractures determined by lateral radiography. There was also a significant relation between the progression of even mild self-reported kyphosis and radiographically detected fractures in our study. It is likely that many elderly people do not accurately remember their height at younger ages. One study of 8610 patients (mean age 70.9 years) noted that the patients’ estimated current height tended to be incorrect, with a mean difference of −2.5 cm from the current measured height^15^. Thus, memory difficulties might have caused the differences of the previous and present research. Also, as a methodology for evaluating OP based on posture, Green *et al*. reported that a wall–occiput distance of >0 cm and a rib–pelvis distance of less than two fingerbreadths suggest the presence of occult spinal fracture^23^. These authors also reported that height loss may be a useful surrogate marker for screening patients with undetected vertebral fractures. These methodologies are easily tested, although each method requires actual measurements of height or distance. In this study, our method required only simple self-reporting of the degree of kyphosis.

It is well known that vertebral fractures likely cause kyphosis. However, generally, it is not rare that vertebrae at the thoracolumbar junction are wedge-shaped, indicating that a patient with wedged-shape vertebrae might feel mild kyphosis. Thus, recognition of kyphosis does not necessarily imply the existence of a fracture. It is therefore challenging to evaluate subjectively whether a patient who feels mild kyphosis is experiencing vertebral fracture—even when using radiographs.

To date, there has been no method for evaluating kyphosis simply; as such, it is difficult to evaluate its severity. The present study showed that there is a significant risk of vertebral fracture even in patients with mild kyphosis. Thus, recognition of kyphosis and recognition of kyphosis severity are different entities. It has been speculated that severity of self-reported kyphosis is associated with the presence of vertebral fractures. Self-evaluation of kyphosis severity may thus be a helpful methodology with which to evaluate the possibility of vertebral fractures being present. Such inquiries would be important with respect to the medical examination and screening of patients at high risk of OP. The results of our current study indicated that self-reported kyphosis could be a useful surrogate marker of vertebral fracture in women aged ≥60 years.

The study showed that there were significant associations between age, current height, and number of fractures. These findings are reasonable because (1) the older the patients are, the more frequent are the fractures they have sustained; and (2) the fewer fractures the patients have, the taller they remain. Other factors contributed less to our findings. Steroid usage, for example, had a negative effect on developing a severe fracture. In fact, not all of the patients on a steroid or who had taken bisphosphonates had a severe fracture. Additionally, the patients given OP medication had less-severe fractures, probably because bisphosphonate treatment is less likely to be associated with severe fractures.

The most serious limitation of this study is that the plain radiographs were not obtained at the exact time of administering the questionnaire. However, before the questionnaires were applied, plain radiographs had been obtained in which we expected an increased frequency of fractures. Thus, we propose that examination of the radiographs and questionnaires be performed at the exact same time, which could increase the validity of the results in this study. Additionally, because we evaluated height loss at the time the questionnaire was administered, the comparisons of height loss and kyphosis would not be changed. Since the subjects knew whether they had a vertebral fracture or not, this could influence on the results of self-reported kyphosis and height loss. Nonetheless, the number of SQ1 fractures was only associated with self-reported kyphosis but not with self-reported height loss.

Second limitation is recall bias, which contributes to inaccuracies in body height at a younger age. When screening patients with undetected OP or vertebral fractures, however, it is impossible to know exactly the body height at younger age. A third limitation is the generalizability of our findings. Our study population consisted of patients who visited an orthopaedic clinic; therefore, our results cannot be applied to the population of Japanese women aged ≥60 years in general. In addition, about 90% of the subjects were already taking OP medications when the study began. However, new vertebral fractures were not observed after that treatment had started. This resource-limited environment might have influenced the findings of our study.

The final limitation is exclusion criteria. The prevalence of patients 40 years and older with OP was 3.4% in men and 19.2% in women in Japan^24^. On the other hand, the prevalence of patients with lumbar spondylosis was larger in men than in women. Although not including men is a weakness of our study, men were excluded because of potential influences by lumbar spondylosis and spinal degeneration.

The strength of our current study is a significant association between the number and presence of SQ1 fractures and self-reported kyphosis. The SQ1 fracture is considered a slight fracture. It is not diagnosed as a fracture when we apply a semi-quantitative diagnostic method in our institution. In this study, we do not inform an OP patient that there is a vertebral SQ1 fracture, so the patients do not know that it is present. Nonetheless, in this study, we found a significant association between the presence and number of SQ-detected fractures and self-reported kyphosis. This result implies that self-reported kyphosis may be a useful screening tool for identifying women aged ≥60 years who have undetected, asymptomatic vertebral fractures.

## Conclusion

To date, there has been no definitive study regarding the association between self-reported kyphosis, self-reported height loss, and the presence and number of undetected vertebral fractures in women aged ≥60 years. The results of this study showed that both self-reported kyphosis and height loss were significantly associated with the presence and number of vertebral fractures in Japanese women aged ≥60 years. These findings imply that these self-reported indices, especially simple self-reporting of kyphosis, may be useful tools for identifying elderly Japanese women who have undetected vertebral fractures.

## Additional Information

**How to cite this article**: Kamimura, M. *et al*. Associations of self-reported height loss and kyphosis with vertebral fractures in Japanese women 60 years and older: a cross-sectional survey. *Sci. Rep.* **6**, 29199; doi: 10.1038/srep29199 (2016).

## Figures and Tables

**Table 1:** Characteristics of 407 study subjects.

Characteristic	Results
Age (years)	72.7 ± 6.4
Height (cm)	150.7 ± 6.1
Weight (kg)	49.9 ± 9.3
Self-reported height loss (cm)	3.4 ± 3.4
Self-reported height loss ≥4 cm	139 (34.2)
Self-reported kyphosis	
None	151 (37.1)
Mild to moderate	207 (50.9)
Severe	49 (12.0)
Current smoking (yes)	4 (1.0)
Previous smoking (yes)	19 (4.7)
Diabetes mellitus (yes)	31 (7.6)
Steroid use (yes)	22 (5.4)
Use of medications for osteoporosis (yes)	366 (89.9)
Rheumatoid arthritis (yes)	14 (3.4)
Fracture (yes)	
All	217 (53.3)
SQ1	144 (35.4)
≥SQ2	127 (31.2)

Results are given as the mean ± SD or number of subjects (%).

**Table 2:** Association between self-reported kyphosis, self-reported height loss, and fracture status.

		Absence of fracture	Presence of fracture
Number of subjects		190	217
Self-reported kyphosis	None	96	55
	Mild to moderate	84	123
	Severe	10	39
Self-reported height loss	<4 cm	151	117
	≥4 cm	39	100

Results are given as number of subjects.

**Table 3:** Associations between the number of vertebral fractures and self-reported kyphosis determined by multiple regression analysis.

Factor		SQ1 fractures	≥SQ2 fractures
Self-reported kyphosis	None	−0.380 ± 0.094	−0.294 ± 0.108
	Severe		0.360 ± 0.161
Age		0.018 ± 0.007	
Self-reported height loss ≥4 cm			0.551 ± 0.123
Use of medication for osteoporosis (yes)			−0.532 ± 0.161
Height (cm)			−0.023 ± 0.009
Steroid use (yes)			−0.483 ± 0.214
*R*^2^		0.067	0.216

Self-reported no kyphosis was significantly associated with a decreased number of SQ1 fractures (p < 0.001). Age was significantly associated with an increased number of SQ1 fractures (p = 0.010). Self-reported no kyphosis (p = 0.007), use of medication for osteoporosis (p = 0.001), height (p = 0.009), and steroid use (p = 0.025) were significantly associated with a decreased number of ≥SQ2 fractures. Self-reported severe kyphosis (p = 0.025) and self-reported height loss ≥4 cm (p < 0.001) was significantly associated with an increased number of ≥SQ2 fractures.

The results are presented as the parameter estimate ± SEM. Parameter estimate is the value for the regression equation for predicting the dependent variable from the independent variable.

SEM, standard error of the mean; SQ, semiquantitative method of grading; self-reported kyphosis consists of three categories (none, mild to moderate, severe).

**Table 4:** Associations between the presence of vertebral fractures and self-reported kyphosis determined by logistic regression analysis with stepwise forward selection.

Factor	Parameter estimate	SEM	Odds ratio	95% CI
SQ1 fractures				
Kyphosis^a^				
1			Reference	
2	1.01	0.24	2.75	1.71–4.43
3	1.27	0.35	3.57	1.80–7.07
Age (year)	0.06	0.02	1.06	1.02–1.10
Height loss (≥ 4 cm)	1.15	0.26	3.16	1.88–5.29
SQ≥2 fractures				
Kyphosis^a^				
1			Reference	
2	0.58	0.28	1.79	1.03–3.11
3	1.24	0.41	3.47	1.56–7.71
OP medications	−1.94	0.57	0.14	0.05–0.43
All fractures				
Height loss (≥4 cm)	0.84	0.24	2.32	1.44–3.74
Kyphosis^a^				
1			Reference	
2	0.76	0.23	2.13	1.36–3.33
3	1.43	0.42	4.17	1.83–9.46

Severity of self-reported kyphosis was significantly associated with an increased risk of SQ1, SQ ≥ 2, and all fractures. Increase of self-reported height loss was significantly associated with an increased risk of SQ1 and all fractures.

SQ, semiquantitative; SEM, standard error of the mean; CI, confidence interval; OP, osteoporosis.

^a^Kyphosis: 1, none; 2, mild to moderate; 3, severe.

Parameter estimate is the value for the logistic regression equation for predicting the dependent variable from the independent variable.
